# Hardiness as a resource of military personnel professional activity

**DOI:** 10.1192/j.eurpsy.2021.1989

**Published:** 2021-08-13

**Authors:** Y. Novikova, D. Boyarinov, L. Gubaidulina, A. Kachina, V. Barabanshchikova

**Affiliations:** Faculty Of Psychology, Lomonosov Moscow State University, Moscow, Russian Federation

**Keywords:** Military Personnel, Resource, hardiness, Chronic Stress

## Abstract

**Introduction:**

The activity of military personnel is associated with risk and tension which can affect both physical and mental health. Hardiness reflects certain characteristics of a person that can motivate them to take an active part in overcoming difficult circumstances. Thou we considering Hardiness is a resource for the reliability of professional activity. The study was supported by the RFBR #19-013-00799.

**Objectives:**

Research of Hardiness as a military personnel professional reliability resource.

**Methods:**

The research involved 315 participants, male. Average age 20.12 years (min – 18, max – 32). The participants completed 3 standardized questionnaires: The Occupational Stress Survey (Leonova, 2006), The 16 PF Questionnaire (rus. version, Kapustina (eds.), 2001), Hardiness Survey (rus. ver. by Leontiev, Rasskazova, 2006).

**Results:**

In our study Hardiness value was above-average (M = 101.3; SD = 15.96). Correlation analysis revealed a direct relationship between Hardiness and “Reliability of professional activity” (M = 0; SD = 1) – Chronic stress, Emotional Stability, Motivational Distortion, Apprehensiveness (p = 0.0001; r = 0.678). It also appeared that Hardiness is a predisposition factor of professional reliability activity (adj. R2=0.539). Correlation analysis also revealed an inverse correlation between Hardiness and Chronic stress (p = 0.0001; r = -0.730).

**Conclusions:**

Thus Hardiness is a resource for the reliability of professional activity. These results can be used in practice for performing trainings to support specialists and help them develop resources for reliability of professional activity.

**Disclosure:**

No significant relationships.

